# Regulation of Glutamate, GABA and Dopamine Transporter Uptake, Surface Mobility and Expression

**DOI:** 10.3389/fncel.2021.670346

**Published:** 2021-04-13

**Authors:** Renae M. Ryan, Susan L. Ingram, Annalisa Scimemi

**Affiliations:** ^1^School of Medical Sciences, Faculty of Medicine and Health, University of Sydney, Sydney, NSW, Australia; ^2^Department of Neurological Surgery, Oregon Health & Science University, Portland, OR, United States; ^3^Department of Biology, SUNY Albany, Albany, NY, United States

**Keywords:** glutamate, GABA, dopamine, transporter, uptake, surface mobility

## Abstract

Neurotransmitter transporters limit spillover between synapses and maintain the extracellular neurotransmitter concentration at low yet physiologically meaningful levels. They also exert a key role in providing precursors for neurotransmitter biosynthesis. In many cases, neurons and astrocytes contain a large intracellular pool of transporters that can be redistributed and stabilized in the plasma membrane following activation of different signaling pathways. This means that the uptake capacity of the brain neuropil for different neurotransmitters can be dynamically regulated over the course of minutes, as an indirect consequence of changes in neuronal activity, blood flow, cell-to-cell interactions, etc. Here we discuss recent advances in the mechanisms that control the cell membrane trafficking and biophysical properties of transporters for the excitatory, inhibitory and modulatory neurotransmitters glutamate, GABA, and dopamine.

## Glutamate Transporters

In addition to being one of the most abundant amino acids and the main excitatory neurotransmitter in the brain, glutamate controls synapse formation, and maturation, acts as an energy substrate for oxidative metabolism, contributes to the antioxidant properties of glutathione, and can be used as a building block for non-ribosomal peptide synthesis and as the precursor for the biosynthesis of the inhibitory neurotransmitter GABA (Sieber and Marahiel, 2005; Brosnan and Brosnan, 2013; Martinez-Lozada et al., 2016; Walker and van der Donk, 2016). In addition, in the developing brain, glutamate guides cell proliferation, migration, differentiation, and survival of neural progenitor cells (Jansson et al., 2013). Because of the plethora of effects that glutamate can exert, its lifetime in the extracellular space needs to be finely controlled through the activity of a family of Na^+^- and K^+^-dependent secondary active transporters. Geneticists, structural biologists, physiologists and clinicians all have their preferred nomenclature to refer to the five known glutamate transporters subtypes, summarized as follows: (i) Slc1a1/SLC1A1/EAAC1/EAAT3; (ii) Slc1a2/SLC1A2/GLT1/EAAT2; (iii) Slc1a3/SLC1A3/GLAST/EAAT1; (iv) Slc1a6/SLC1A6/EAAT4; (v) Slc1a7/SLC1A7/EAAT5. The use of each terminology is physiologically meaningful, as it refers to the genes encoding each transporter in rodents (Slc1a1, Slc1a2, Slc1a3, Slc1a6, Slc1a7) and in humans (SLC1A1, SLC1A2, SLC1A3, SLC1A6, SLC1A7), or the protein product in rodents (EAAC1, GLT1, GLAST, EAAT4-5) and humans (EAAT1-5). For simplicity, in this review, we refer to them as EAAT1-5. Different subtypes of glutamate transporters are differentially expressed in neuronal and glial cells (Danbolt, 2001). Accordingly, the glutamate transporters EAAT1-2 are mostly expressed in astrocytes, whereas EAAT3-5 are mostly expressed in neurons. Two of the neuronal transporters are predominantly expressed in the cerebellum (EAAT4) or retina (EAAT5), with a low yet experimentally measurable concentration in the forebrain (Dehnes et al., 1998; Danbolt, 2001). The others (i.e., EAAT1, EAAT2, and EAAT3) are expressed broadly throughout the brain, at varying levels.

**FIGURE 1:**
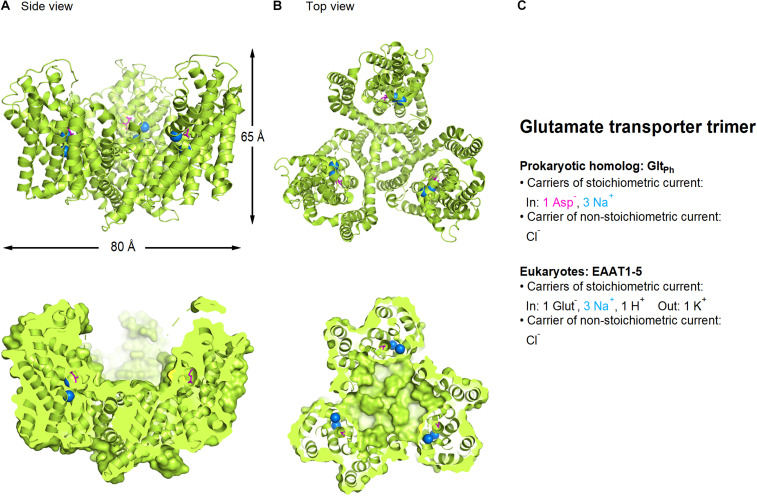
Crystal structure of Glt_*Ph*_, a bacterial homolog of glutamate transporters (PDB: 2NWX). **(A)** Side view of the glutamate transporter trimer parallel to the membrane, using a ribbon (*top*) and a surface representation sliced through the center of the transporter basin (*bottom*). **(B)** As in A, viewed from the extracellular side of the membrane. Aspartate (*magenta*) and sodium ions (*blue*) are represented as spheres. **(C)** Summary of the main feature of the stoichiometric and non-stoichiometric current mediated by glutamate transporters in prokaryotes and eukaryotes, respectively. Image generated using The PyMOL Molecular Graphics System (Version 2.3.2; Schrödinger, 2010).

Structural information of the glutamate transporters comes from prokaryote homologs such as Glt_*Ph*_ and Glt_*Tk*_ which share ∼35 amino acid identity with human EAAT2 (Yernool et al., 2004; Boudker et al., 2007; Reyes et al., 2009; Verdon and Boudker, 2012; Guskov et al., 2016; Scopelliti et al., 2018) and more recent crystal and cryo-EM structures of human transporters including EAAT1, EAAT3, and ASCT2 (Canul-Tec et al., 2017; Garaeva et al., 2018; Garaeva et al., 2019; Yu et al., 2019; Wang and Boudker, 2020) (Figure 1). All members of this family appear to assemble as trimers, with each monomer capable of transporting substrate and coupled ions, generating stoichiometric and non-stoichiometric currents, independently of the two other monomers (Grewer et al., 2005; Koch et al., 2007; Leary et al., 2007). The transporters are composed of a “transport domain” which binds and transports substrate and coupled ions, and a “scaffold domain” that forms inter-protomer contacts and interacts with the lipid membrane (Boudker et al., 2007; Reyes et al., 2009). Glt_*Ph*_ transports aspartate together with three Na^+^ ions into the cytoplasm using a twisting elevator mechanism (Reyes et al., 2009; Ryan and Vandenberg, 2016) and generates a stoichiometrically uncoupled Cl^–^ conductance (Boudker et al., 2007; Ryan and Mindell, 2007; Reyes et al., 2009).

In contrast to prokaryotes, for all eukaryotic transporters, glutamate uptake is driven by the electrochemical gradient for Na^+^ and H^+^ ions, and the rate limiting step is the counter-transport of one K^+^ ion across the membrane (Zerangue and Kavanaugh, 1996). The stoichiometry of the transport process is the inward movement of 1 glutamate (which carries a negative charge): 3 Na^+^: 1 H^+^, followed by the counter-transport of 1 K^+^, leading to the net influx of two positive charges per transport cycle (Zerangue and Kavanaugh, 1996; Levy et al., 1998). In addition, these transporters mediate a glutamate-dependent anion flux, which under physiological conditions is carried by Cl^–^ ions (Picaud et al., 1995; Wadiche et al., 1995b; Eliasof and Jahr, 1996). The chloride channel forms at the interface of the transport and scaffold domains during the transport cycle (Ryan et al., 2004; Cater et al., 2014, 2016; Cheng et al., 2017; Kolen et al., 2020; Chen et al., 2021) and the direction of this flux is determined by the driving force for chloride (i.e., the difference between membrane potential and reversal potential for chloride). The function of the chloride current remains incompletely understood but it has been suggested that it might serve to counterbalance the influx of positive charges due to glutamate transport and prevent cell depolarization (Grewer and Rauen, 2005). This hypothesis would only hold true in the presence of a positive driving force for chloride, causing an inward movement of chloride into the cell. This occurs when the reversal potential for chloride is more hyperpolarized than the membrane potential. This condition, however, is not fulfilled in developing neurons. Here, the reversal potential for chloride is more depolarized than in mature neurons, due to a delayed expression of the potassium chloride co-transporter KCC2, and a consequently higher intracellular chloride concentration in developing compared to mature neurons (Rivera et al., 1999). Other physiological roles for the chloride conductance have been demonstrated with EAAT5 in the retina, where it regulates cell activity and neurotransmitter release (Picaud et al., 1995; Veruki et al., 2006) and in cerebellar astrocytes, where the chloride currents of EAAT1/2 affects the resting intracellular chloride concentration (Sonders and Amara, 1996; Untiet et al., 2017).

### Similarities and Distinctive Biophysical Features of Different Glutamate Transporter Subtypes

The general mechanisms of substrate transport, identified through studies of uptake of radiolabeled substrates or voltage-clamp recordings of transporter currents under steady-state conditions, are roughly similar among different glutamate transporter types. For all of them, the transport efficiency is in the order of ∼50%, which means that − perhaps surprisingly − these molecules translocate into the cell cytoplasm only 50% of all glutamate molecules they bind (Tzingounis and Wadiche, 2007). The remaining 50% of glutamate molecules that unbind from the transporters are released back in the extracellular space, and ultimately bind to nearby glutamate receptors, neuronal or glial transporters. This low transport efficiency can lead to an apparently paradoxical prolongation of glutamate lifetime in the extracellular space (Scimemi et al., 2009). Since the rates of glutamate binding to receptors and transporters are relatively similar, the likelihood that a glutamate molecule unbound from a transporter will eventually bind to a receptor or another transporter depends on the relative abundance of receptors compared to that of transporters. Typically, the surface density of expression of transporters in glial membranes is four orders of magnitude higher than that of glutamate receptors in extra-synaptic neuronal membrane (Lehre and Danbolt, 1998). For this reason, the most likely ultimate fate of glutamate molecules unbound from transporters is to be bound again by other transporter molecules (Leary et al., 2011).

Non-rate-limiting partial reaction steps, obtained by perturbing the steady-state and by subsequently following the kinetics of the relaxation to a new steady-state (i.e., the pre-steady-state kinetics), are similar for EAAT1-3 (Grewer and Rauen, 2005). However, the pre-steady-state kinetics and steady-state turnover rats are significantly slower for EAAT4 (Mim et al., 2005). There are also notable differences in the steady state affinity of different transporter types (*k*_*m*_ EAAT1: 48 ± 10 μM; EAAT2: 97 ± 4 μM; EAAT3: 62 ± 8 μM; EAAT4: 0.6 μM; Wadiche and Kavanaugh, 1998; Grewer et al., 2000; Bergles et al., 2002), and in the ratio of substrate transport versus anion permeation (Arriza et al., 1994; Seal and Amara, 1999; Mim et al., 2005; Torres-Salazar and Fahlke, 2007). Interestingly, and in contrast to EAAT1-3, the apparent affinity for glutamate is voltage dependent for EAAT4 and increases with negative voltages, suggesting higher glutamate buffering capacity for EAAT4 than other glutamate transporters (Mim et al., 2005). The fact that EAAT4 has a 10-fold higher affinity for glutamate but a 10-fold slower translocation rate than other transporters has led to hypothesize that the main functional role of EAAT4 is accounted for by its ability to generate a stoichiometrically uncoupled anion current (Fairman et al., 1995; Lin et al., 1998). Others have suggested that these biophysical properties would allow EAAT4 to clear glutamate away from synapses, where its concentration is lower than at the outer boundary of the synaptic cleft (Mim et al., 2005). Consistent with this hypothesis, one of the most prominent roles of EAAT4 is to limit metabotropic glutamate receptor activation in cerebellar Purkinje cells, in sub-cellular domains where the density of expression of these receptors and EAAT4 are both high (Wadiche and Jahr, 2005).

Glutamate transport via EAAT4 has a unique voltage-dependence. Its maximum transport activity is detected at –20 mV < V_*m*_ < 0 mV and the transporter inactivates at more negative membrane potentials (Mim et al., 2005). Membrane hyperpolarization promotes glutamate transport via other glutamate transporters, which have reversal potentials of 9.3 ± 0.7 mV (EAAT1), >80 mV (EAAT2) and 38.0 ± 2.7 mV (EAAT3) (Arriza et al., 1994). At hyperpolarized potentials, not only transport, but also the anion conductance of EAAT4 is inhibited (Mim et al., 2005). This means that at membrane potentials close to the resting potential of neurons, glutamate is bound strongly to all transporters, but its transport via EAAT4 is inhibited (Mim et al., 2005).

There are differences in the sodium requirement for activation of the anion conductance between neuronal and glial glutamate transporters (Wadiche et al., 1995a; Grewer et al., 2000, 2001; Otis and Kavanaugh, 2000). For EAAT3, the anion conductance can be activated by glutamate and Na^+^ ions from both sides of the membrane (Watzke and Grewer, 2001). The activation of the anion conductance by sodium alone has only been demonstrated for EAAT3-4, while EAAT1-2 mediate glutamate- and sodium-independent anion conducting states (Divito et al., 2017). For EAAT4-5, the anion conductance is particularly large compared to their glutamate transport capacity (Sonders and Amara, 1996; Seal et al., 2001). Consequently, transport currents generated by EAAT1-3 are easily measured experimentally using heterologous expression systems, whereas those mediated by EAAT4-5 are relatively small (Wadiche et al., 1995a; Grewer et al., 2001; Mitrovic et al., 2001; Watzke et al., 2001).

The existence of functional differences in the properties of glutamate transporter subtypes indicates that the function of these molecules is much more complex than previously thought, and that an evaluation of the physiological implications of glutamate transporters cannot bypass an understanding of the biophysical properties of these molecules in their native environments.

### The Surface Mobility and Cellular Distribution of EAAT2

Out of all glutamate transporter types, EAAT2 has the highest density of expression in the adult brain, and is responsible for the largest proportion of glutamate transport (Minelli et al., 2001). In astrocytes, 70–75% of all EAAT2 is expressed on the plasma membrane (Michaluk et al., 2020). In the hippocampus, cerebellum and neocortex, EAAT2 is expressed mostly in astrocytic processes, in the vicinity and at a distance from the synaptic cleft (Danbolt et al., 1992; Levy et al., 1993; Rothstein et al., 1994; Torp et al., 1994; Chaudhry et al., 1995; Lehre et al., 1995). EAAT2 exists in at least three splice variants which differ only in their C-terminal domain. EAAT2a is the predominant variant, and represents ∼90%, whereas EAAT2b and EAAT2c represent ∼6% and ∼1% of total EAAT2 protein, respectively (Chen et al., 2002; Rauen et al., 2004; Holmseth et al., 2009). These variants have similar regional distribution and although they are all primarily expressed in astrocytes, they have also been detected in neurons (Schmitt et al., 2002; Chen et al., 2004; Maragakis et al., 2004; Bassan et al., 2008; Gonzalez-Gonzalez et al., 2008a; Holmseth et al., 2009). EAAT2a is more abundantly expressed than EAAT2b, and is a presynaptic glutamate transporter (Berger et al., 2005; Rimmele and Rosenberg, 2016). Within the hippocampus, 14–29% of axon terminals express EAAT2a (Chen et al., 2004). Though EAAT2a and EAAT2b share similar functional properties, they differ for the sequence of their extreme intracellular C-terminus (Chen et al., 2002; Sullivan et al., 2004). Unlike EAAT2a, EAAT2b has a PDZ binding domain that makes it capable of binding to proteins like PICK1, PSD95, and DLG1 (Bassan et al., 2008; Gonzalez-Gonzalez et al., 2008a; Underhill et al., 2015). These, in turn, can alter the currents mediated by the EAAT2b transporter (Sogaard et al., 2013). EAAT2a forms heteromers with EAAT2b, which have been proposed to stabilize EAAT2a around synapses (Haugeto et al., 1996; Peacey et al., 2009).

EAAT2 is highly mobile on the plasma membrane of astrocytes (*D^∗^* = 0.15–0.23 μm^2^/s) (though a lower value of *D^∗^* = 0.039 μm^2^/s has been measured using quantum dots; Al Awabdh et al., 2016), and the rate with which it diffuses along the plasma membrane can be increased by glutamate binding to EAAT2 or to AMPA, NMDA and metabotropic glutamate receptors, and can be reduced by blocking glutamate binding to EAAT2 (Benediktsson et al., 2012; Murphy-Royal et al., 2015; Michaluk et al., 2020). The surface diffusion of the EAAT2 variants EAAT2a and EAAT2b is more confined at astrocytic processes proximal to synapses, especially for EAAT2b (Al Awabdh et al., 2016). Consistent with previous work, glutamate increases the surface mobility of both EAAT2 variants, whereas blocking synaptic activity reduces it (Al Awabdh et al., 2016). The more confined diffusion of EAAT2b may be attributed to the presence of the PDZ domain, which allows EAAT2b to interact with scaffolding proteins and anchor it to macromolecular complexes that astrocytes form in subcellular domains opposite to neuronal presynaptic terminals (Al Awabdh et al., 2016). There is also evidence that membrane raft association regulates the targeting and function of glutamate transporters, especially for EAAT2 (Butchbach et al., 2004).

Glutamate transporters have been suggested to become incorporated into the plasma membrane through exocytosis (Cheng et al., 2002; Chowdhury et al., 2002; Robinson, 2002; Fournier et al., 2004; Karylowski et al., 2004; Zeigerer et al., 2004), and several components of this molecular machinery have been identified in astrocytes (Parpura et al., 1995; Hepp et al., 1999; Zhang et al., 2004). Consistent with these findings, calcium-dependent exocytosis of EAAT2 has been detected using FM dyes in cultured astrocytes (Stenovec et al., 2008). The same experiments showed that EAAT2 has a punctate distribution along the astrocytic plasma membrane, suggesting that the glutamate uptake capacity of these cells varies depending on the local density of expression of glutamate transporters (Stenovec et al., 2008). Interestingly, recent work indicate that the lifetime of EAAT2 on the astrocyte plasma membrane is ∼22 s (Michaluk et al., 2020). Whereas lateral diffusion could serve as a mechanism for transporter turnover away from synapses, where the diffusivity of these molecules is higher, endo/exocytosis from/to the plasma membrane to intracellular organelles could contribute to transporter turnover in astrocytic processes nearby synapses, where the diffusivity of these molecules is lower (Michaluk et al., 2020).

Although 80–90% of EAAT2 is localized in astrocytes, there is a small proportion (5–10%) in neuronal axon terminals (Rauen and Kanner, 1994; Torp et al., 1994; Schmitt et al., 1996; Torp et al., 1997; Chen et al., 2004; Furness et al., 2008; Melone et al., 2009, 2011, 2019). In neurons, EAAT2 co-localizes with the α_1_ and α_3_ isoforms of the Na^+^/K^+^-ATPase, but this co-localization is looser than that with the α_2_ isoform in astrocytes, suggesting a less efficient interaction between EAAT2 and the Na^+^/K^+^-ATPase in neurons (Melone et al., 2019). Neuronal EAAT2 appears to be required to provide glutamate to synaptic mitochondria, and is therefore linked to energy metabolism (Petr et al., 2015; Fischer et al., 2018; McNair et al., 2019, 2020; Sharma et al., 2019). By contrast, astrocytic EAAT2 is crucial to ensure survival, resistance to epilepsy, and prevent cognitive decline. Loss of neuronal and astrocytic EAAT2 have both implications on long-term memory and spatial reference learning, but the time scale over which they exert these effects is different (Sharma et al., 2019). Loss of astrocytic EAAT2 leads to early deficits, whereas loss of neuronal EAAT2 leads to late-onset deficits in long-term memory and spatial reference learning (Sharma et al., 2019). These findings are important because they identify neuronal and astrocytic EAAT2 as contributing to different aspects of cognitive function and, potentially, as different therapeutic targets in cognitive decline (Petr et al., 2015; Fischer et al., 2018; McNair et al., 2019, 2020; Sharma et al., 2019).

Multiple studies have shown that there are interesting relationships between astrocytic coverage, glutamate transporter expression and synaptic size (Ventura and Harris, 1999; Genoud et al., 2006; Witcher et al., 2007; Lushnikova et al., 2009; Witcher et al., 2010; Patrushev et al., 2013; Medvedev et al., 2014; Gavrilov et al., 2018; Herde et al., 2020). For example, in the rodent hippocampus, only 40–60% of synapses have astrocytic processes, and these cover only 53% of their perimeter (Ventura and Harris, 1999; Witcher et al., 2007, 2010). The size of a spine correlates with its release probability (Schikorski and Stevens, 1997). Astrocytes are closer to smaller spines (Medvedev et al., 2014), but the overall astrocytic coverage does not change with spine size (Gavrilov et al., 2018). Although there is less EAAT2 at smaller spines, its density is higher (Herde et al., 2020). These findings have important implications for understanding how glutamate transporters control spillover at synapses with different size (Scimemi et al., 2004), and the implications of this phenomenon on synaptic and astrocyte plasticity (Wenzel et al., 1991; Matsusaki et al., 2001; Bernardinelli et al., 2014; Perez-Alvarez et al., 2014).

### Transcriptional, Translational, and Post-translational Regulation of Glutamate Transporters

Despite current agreements on the presence of EAAT2 in neurons, in the 1990s, some groups failed to find EAAT2 proteins in subsets of cortical neurons (Rothstein et al., 1994; Chaudhry et al., 1995; Lehre et al., 1995), in contrast to others (Torp et al., 1994; Schmitt et al., 1996; Torp et al., 1997). The discrepancy between these findings was attributed to the existence of a post-transcriptional and post-translational control of EAAT2 expression (Gegelashvili and Schousboe, 1997; Anderson and Swanson, 2000; Plachez et al., 2000). In fact, like most membrane proteins, glutamate transporters can be regulated at the gene expression, protein targeting and trafficking, and post-translational level.

The gene structure and organization of most glutamate transporters identified in the late 1990s showed that the promoter regions are highly conserved between mouse and human (Hagiwara et al., 1996; Stoffel et al., 1996). These regions do not contain a TATA box but a GC box and, in humans, an E box (Martinez-Lozada et al., 2016). The genes encoding different glutamate transporters contain different excision/splicing sites for different exons, which can generate variant mRNA species and proteins. Accordingly, multiple studies have reported the existence of several mRNA size classes encoding EAAT1 and EAAT3 (Pines et al., 1992; Tanaka, 1993; Mukainaka et al., 1995; Nakayama et al., 1996; Palos et al., 1996; Gegelashvili and Schousboe, 1997). This might be due to differences in polyadenylation and the existence of mRNAs with different coding capacity (Gegelashvili and Schousboe, 1997).

When glutamate transporters and their human homologs were first cloned, knowledge on the elements regulating their transcription largely relied on information collected from ASCT1, a neutral amino acid transporter of the same family of high affinity transporters as glutamate transporters (Hofmann et al., 1994). ASCT1 has 39–44% amino acid sequence identity, similar hydropathy profiles, *trans*-membrane organization and conservation of crucial function-related motifs compared to EAAT2 (Gegelashvili and Schousboe, 1997). According to these initial reports, the promoter region of EAAT2 was thought to contain at least five consensus sequences for the *Sp1* transcription factor (*krox24, krox20, Egr3, NGFI-C)*, involved in cell differentiation (Hofmann et al., 1994; Gegelashvili and Schousboe, 1997). By the early 2000’s, cloning and bioinformatics works established that the promoter region for EAAT1-2 is highly conserved: not only does it not have a TATA box, but lacks well-defined *cis-*elements and contains five consensus sequences for *Sp1* and GC-rich repeats, also found in humans (Su et al., 2003). The search for regulatory transcription factors has led to the identification of the Nuclear Factor of Activated T cells (NFAT), the *N*-myc proto-oncogene protein (*N*-myc), and the Nuclear Factor κB (NF-κB), and a consensus NF-κB binding sequence in the 5′-UTR region of the *Slc1a2* gene in humans (Meyer et al., 1996; Su et al., 2003). The Tumor Necrosis Factor α (TNFα), a cytokine involved in the acute phase of inflammatory reactions, decreases EAAT2 mRNA expression by increasing NF-κB activation (Su et al., 2003), perhaps by promoting NF-kB interactions with other transcription factors (Sitcheran et al., 2005). The ability of transcription factors to regulate gene expression can change the sensitivity of glutamate transporter expression to other regulatory proteins. For example, TNFα regulates the activity of the Yin Yang 1 (YY1) transcription factor which, when bound to the EAAT2 promoter, changes the effect of NF-κB from activation to suppression (Karki et al., 2014). In turn, NF-κB regulates YY1 expression. These findings suggest the existence of complex interplays between NF-κB and YY1 for the transcriptional regulation of EAAT2 expression.

Over the last two decades, other groups have identified factors, cis-regulatory elements and epigenetic mechanisms regulating EAAT1-2 transcription, but many unknowns remain about the molecular mechanisms regulating EAAT3-5 expression. In Table 1, we provide a summary of current studies on modulation of glutamate transporters listed in PubMed. One can easily note that in many cases, the results are conflicting. One may argue that inconsistencies are inevitable when studying a given transporter in different cell types and animal species. However, in some cases these considerations do not allow to resolve conflicting results from different laboratories. For this reason, in this review, we limit our discussion to forms of modulation of glutamate transporters for which some consensus exists.

**TABLE 1:** Regulating factors of glutamate transporter uptake and expression.

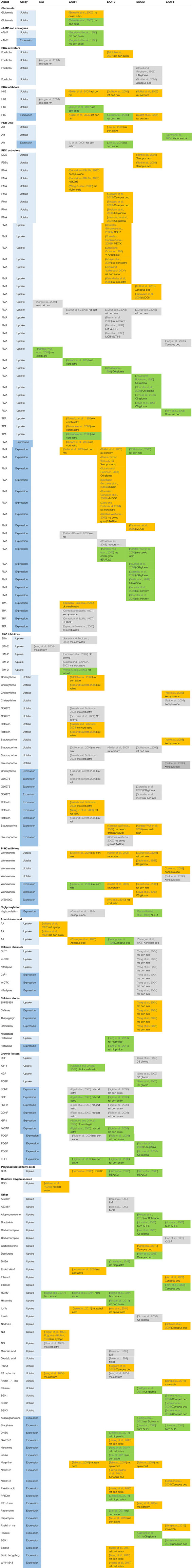

*The table provides a summary of the results obtained in the literature regarding the mechanisms controlling the rate of glutamate uptake and the expression of glutamate transporters in a variety of animal models and expression systems. The cells in the first column of the table are color-coded to highlight studies focusing on glutamate uptake (light blue) or glutamate transporter expression (dark blue). The third column, labeled N/A, refers to studies that did not distinguish among different glutamate transporter subtypes. The cells in the last four columns are color-coded to highlight increases (green), decreases (orange), or no change (gray) in glutamate transporter uptake or expression. Abbreviations: mouse (ms), human (hum), cortex (cort), cerebellum (cereb), hippocampus (hipp), spinal cord (spin cord), astrocyte (astro), neuron (nrn), granule cell (gran), Schwann cells (Schwann), oocyte (ooc), retina (ret), retinoblastoma (retblast), synaptosome (synapt), arachidonic acid (AA), Bisindolylmaleimide (BIM), ω-conotoxin (ω-CTX), dehydroepiandrosterone (DHEA), phorbol 12-myristate 13-acetate (PMA), and 12-0-tetradecanoylphorbol-13-acetate (TPA).*

There is a general agreement on the fact that in cultured neurons, PACAP, cAMP, and PKA-dependent pathways increase EAAT1 expression (Martinez-Lozada et al., 2016) and uptake (Hertz et al., 1978; Gegelashvili et al., 1996). Presumably this effect is mediated by activation of the transcription factor cAMP-response element binding protein (CREB), but the transcription factors or the *cis* elements of the promoter responsible for this effect have not been identified (Martinez-Lozada et al., 2016). This form of transcriptional regulation may provide a pathway through which activation of G-protein membrane receptors coupled to cAMP and PKA-dependent signaling pathways indirectly affect glutamate uptake, as it has been shown in other contexts for D_2_ dopamine receptors or α_1_ and β-adrenergic receptors (Kerkerian et al., 1987).

Stable epigenetic alterations of glutamate transporter gene expression are heritable in the short term, but do not involve DNA mutations. These include methylation and histone modifications, and there are multiple CpG regions in the promoter for EAAT2 where methylation can occur (Zschocke et al., 2005). One of the consequences of methylation is that it alters the ability of glucocorticoids to change EAAT2 expression. Accordingly, in the cerebellum, where the EAAT2 promoter is hyper-methylated, glucocorticoids are unable to change EAAT2 expression (Zschocke et al., 2005). By contrast, in the forebrain, where the EAAT2 promoter is hypo-methylated, glucocorticoid up-regulate EAAT2 expression (Zschocke et al., 2005).

Phosphorylation and glycosylation are two documented forms of post-translational modifications for glutamate transporters (Gegelashvili and Schousboe, 1997). Accordingly, increasing protein kinase C (PKC) activation by phorbol esters leads to increased phosphorylation of EAAT2 at the residue Ser113 and increased glutamate uptake (Roginski et al., 1993). *N-*glycosylation promotes EAAT3 expression, but has no effect on EAAT1 (Conradt et al., 1995; Ferrer-Martinez et al., 1995).

Arachidonic acid, produced via activation of NMDA receptors in neurons and metabotropic glutamate receptors in astrocytes, can directly interact with different types of glutamate transporters, with different effects. For example, it reduces glutamate uptake via EAAT1, enhances glutamate uptake via EAAT2 and leads to a moderate increase of glutamate uptake via EAAT3 (Chan et al., 1983; Volterra et al., 1992; Trotti et al., 1995). Arachidonic acid can lead to the activation of PKC, promoting glutamate uptake via EAAT2. Metabolites of arachidonic acid can be a source of reactive oxygen species (ROS), which inhibit glutamate uptake but increase the transporters’ steady state affinity for glutamate (Volterra et al., 1994b).

There is a growing awareness that these forms of regulation are complex not only because they likely differ among species, cell types and brain regions, but also because they interact with one another in ways that can be difficult to reproduce in reduced preparations but that affect the function of these transporters *in vivo*. These currently unknowns are likely going to be addressed by using experimental approaches that allow manipulation of different regulation factor *in situ*, and in a cell-specific manner.

### Activity-Dependent Modulation of Glutamate Transporter Trafficking

The first observation that glutamate itself can modulate its uptake came from data showing that glutamate uptake is increased in astrocyte cultures supplemented with conditioned media from neuronal cultures (Drejer et al., 1983; Voisin et al., 1993). Pure astrocytic cultures only express EAAT1, but co-culture of neurons and astrocytes increases EAAT1 expression and induces EAAT2 expression (Gegelashvili and Schousboe, 1997; Swanson et al., 1997). This effect depends on p42/44 MAP kinases activation via the tyrphostin-sensitive Receptor Tyrosine Kinase (RTK) signaling pathway, and is abolished by inhibitors of PI3K, tyrosine kinase and NF-kB (Swanson et al., 1997; Zelenaia et al., 2000). Similarly, decreased glutamate uptake via EAAT1-2 (not EAAT3) occurs in response to axotomy of glutamatergic neurons of cortical lesions (McGeer et al., 1977; Shifman, 1991; Ginsberg et al., 1995; Levy et al., 1995). Together, these forms of activity-dependent regulation of EAAT2 expression allow this transporter to be more abundant and less mobile in astrocytic processes close to active glutamatergic synapses, an effect that can provide an effective strategy to limit glutamate spillover away from the synaptic cleft.

Similarly, to glutamate, glutamate transporter substrates like D-Aspartate and ligands like L-*trans*-PDC and TBOA can also produce a redistribution of EAAT1 on the cell membrane (Shin et al., 2009). Consistent with these findings, treating astrocyte cultures with kainate, dbcAMP or AMPA receptor agonists increases D-aspartate uptake and EAAT1 protein expression (Gegelashvili et al., 1996). It is unclear whether these effects are due to binding to AMPA receptors or are due to release of diffusible molecules (e.g., arachidonic acid, diacylglycerol, nitric oxide) through more complex intracellular signaling cascades (Gegelashvili and Schousboe, 1997).

### Physiological Roles of Glutamate Transporters

The role of glutamate transporters in regulation of phasic and tonic extracellular glutamate levels is critical for neuronal signaling and controlling excitotoxicity (Rothstein et al., 1996). Spillover of glutamate and inter-synaptic cross-talk have been associated with multiple neuropsychiatric disorders, including schizophrenia, epilepsy, addiction, depression and obsessive compulsive disorder (O’Donovan et al., 2017; Bellini et al., 2018; Malik and Willnow, 2019). Knock-out mouse models of different subtypes of glutamate transporters have revealed major motor deficits with decreased levels of glial transporters EAAT1-2 (Rothstein et al., 1996), with more subtle behavioral deficits with loss of the neuronal transporter EAAT3 (Peghini et al., 1997; Bellini et al., 2018).

In humans, decreased expression and reduced function of EAAT2 are associated with amyotrophic lateral sclerosis (ALS) (Rothstein et al., 1995; Bruijn et al., 1997; Rosenblum and Trotti, 2017). In transgenic mice expressing an N-terminal fragment of mutant huntingtin (R6/2), there is an age-dependent downregulation of EAAT2, which leads to a progressive increase in the extracellular glutamate (Behrens et al., 2002). Other glutamate transporters, however, remain unchanged, suggesting that EAAT2-mediated excitotoxicity might contribute to Huntington’s disease (Behrens et al., 2002). In the few identified polymorphisms of the gene encoding EAAT3, dicarboxylic aminoaciduria, a deficit in kidney function was prominent, as well as family linkage to schizophrenia and obsessive–compulsive disorders (OCD) (Bailey et al., 2011; Porton et al., 2013). In mice, loss or increased EAAT3 are associated with increased anxiety and OCD-like behaviors, suggesting that preserving an optimal expression of this transporter is key for the function of neuronal circuits affected by this disease (Zike et al., 2017; Bellini et al., 2018; Delgado-Acevedo et al., 2019). Significant lower levels of EAAT2 have also been reported *in vitro* (Scimemi et al., 2013) and *in vivo*, in animal models of Alzheimer’s disease (Wilson et al., 2003; Dabir et al., 2006; Mookherjee et al., 2011; Schallier et al., 2011; Hefendehl et al., 2016), as well as in humans affected by this disease (Li et al., 1997; Jacob et al., 2007; Scott et al., 2011; Leng et al., 2021), whereas promoting EAAT2 expression improves cognitive function (Fan et al., 2018). Several mutations in the gene that encodes EAAT1 have also been linked to the neurological disease Episodic Ataxia Type 6 (Choi et al., 2017; Chivukula et al., 2020). The most well-studied mutation (P290R) results in reduced glutamate transport activity and a large increase in the uncoupled anion conductance, the latter property being suggested to be responsible for the phenotype in studies using a *Drosophila melanogaster* model (Winter et al., 2012; Parinejad et al., 2016). Further understanding of how these transporters are regulated in specific circuits and synapses may allow for design of therapeutics that correct for specific deficits in transporter function.

## GABA Transporters

**FIGURE 2:**
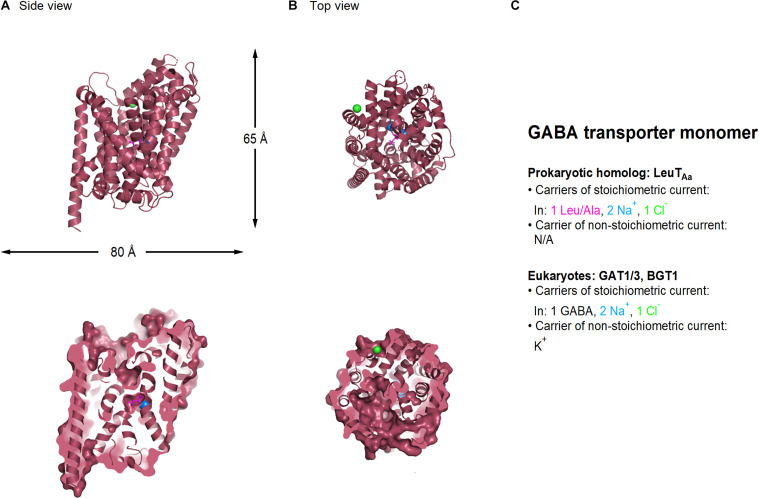
Crystal structure of LeuT_*AA*_, a bacterial homolog of GABA transporters (PDB: 2A65). **(A)** Side view of the LeuT_*Aa*_ transporter parallel to the membrane, using a ribbon (*top*) and a surface representation sliced through the center of the transporter (*bottom*). **(B)** As in **(A)**, viewed from the extracellular side of the membrane. Leu (*magenta*), sodium (*blue*), and chloride ions (*green*) are represented as spheres. **(C)** Summary of the main feature of the stoichiometric and non-stoichiometric current mediated by Leu and GABA transporters in prokaryotes and eukaryotes, respectively. Image generated using The PyMOL Molecular Graphics System (Version 2.3.2; Schrödinger, 2010).

In mammals, GABA transporters are classified into four subtypes, based on amino acid sequence homology and pharmacological properties. These include GAT1-3 and the betaine GABA transporter BGT1. Out of these, GAT1 and GAT3 account for the largest proportion of GABA uptake in the CNS, and for this reason, they will be the focus of our attention in this review. Structural information of the GABA transporters comes from prokaryote homologs such as LeuT_*Aa*_ (Figure 2; Yamashita et al., 2005). In the neocortex, GAT1 is expressed robustly in GABAergic axon terminals, astrocytic processes, oligodendrocytes and microglial cells (Fattorini et al., 2020). The expression of GAT1 is pronounced in the axon terminal of chandelier GABAergic neurons (known as “cartridges”), which provide inhibitory inputs to the axon initial segment of pyramidal cells (Woo et al., 1998). In young mice (P9), GAT1 is also transiently expressed somatically, but this somatic expression is lost in juvenile mice (P29) (Yan et al., 1997; Yan and Ribak, 1998). Presynaptic boutons in the cerebellum and hippocampus express 800–1,300 μm^–2^ GAT1 molecules, with a preferential perisynaptic localization (Chiu et al., 2002; Melone et al., 2015). These density values drop to 640 μm^–2^ GAT1 molecules along the length of the axon (Chiu et al., 2002), whereas the surface density of GAT1 in astrocytic membranes is 3.5 times higher than in axon terminals (Melone et al., 2015). GAT3 is mainly localized in peri-synaptic astrocytic processes, but has also been detected in brainstem and cortical neurons (Clark et al., 1992; Melone et al., 2003, 2005, 2015). These findings are important because they provide anatomical evidence to the fact that no GABA transporter can be described as being purely neuronal or glial, although they may have a preferential distribution in a given cell type depending on the age and brain region of different animal models.

Both GAT1 and GAT3 transporters translocate the zwitterion GABA across the membrane by coupling its movement to the co-transport of two Na^+^ and one Cl^–^ ion, leading to the net influx of one positive charge per transport cycle (1 GABA: 2 Na^+^: 1 Cl^–^) (Radian and Kanner, 1983; Keynan and Kanner, 1988). In addition to this stoichiometric current, GABA transporters mediate an agonist-independent leak current carried by alkali ions, which can be detected in mammalian expression systems but not in *Xenopus laevis* oocytes (Cammack and Schwartz, 1996; Eckstein-Ludwig et al., 1999; Lu and Hilgemann, 1999; MacAulay et al., 2002; Matthews et al., 2009). This current can generate a local change in membrane voltage and/or membrane resistance, which can be more or less pronounced depending on the magnitude and direction of the driving force for the permeant ions and the local density of the transporters. Changes in membrane resistance are important because they act as a local shunt capable of hampering action potential propagation and cell excitability.

As in the case of glutamate transporters, we provide a summary of current studies on modulation of GABA transporters listed in PubMed (Table 2).

**TABLE 2:** Regulating factors of GABA transporter Uptake and expression.

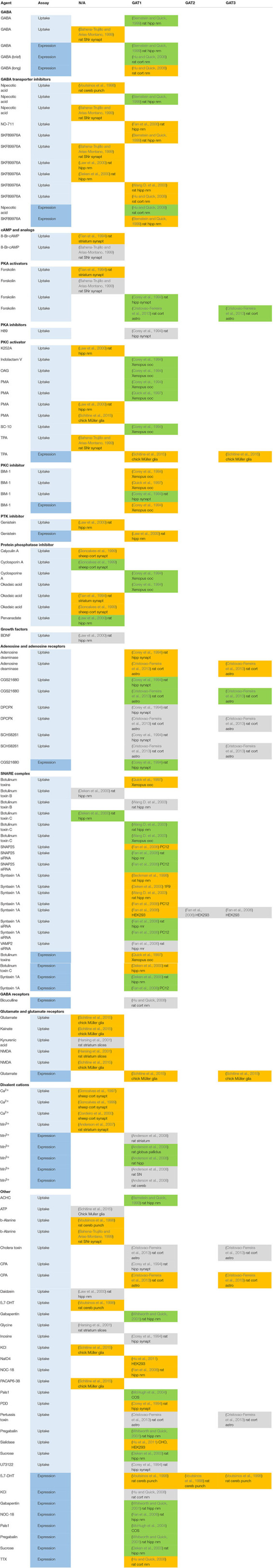

*The table provides a summary of the results obtained in the literature regarding mechanisms controlling the rate of GABA Uptake and its expression in a variety of animal models and expression systems. The color coding of each cell and abbreviations are as described for Table 1. *SNr*, *substantia nigra pars reticulata; ACHC*, *cis*-1,3-aminocyclohexane carboxylic acid; CPA, α-cyclopiazonic acid; *5,7-DHT*, 5,7-dihydroxytryptamine; *OAG*, oleyl-acetyl glycerol; *PDD*, 4 alpha-phorbol-12,13-didecanoate.*

### The Surface Mobility and Cellular Distribution of GAT1

The GABA transporter GAT1 has a membrane pool of 61–63% (Chiu et al., 2002). Fluorescence Recovery After Photobleaching (FRAP) experiments in neuroblastoma 2a cells show that 50% of membrane GAT1 is immobile, likely due to the existence of tight interactions between GAT1 and the actin cytoskeleton, mediated by the adaptor protein ezrin (Imoukhuede et al., 2009). Accordingly, depolymerizing actin or interrupting the GAT1 PDZ-interacting domain increases the transporter mobility, whereas depolymerizing microtubules does not (Imoukhuede et al., 2009). Numerous mechanisms contribute to cycle this pool of transporter to and from the plasma membrane. Specifically, there are two regions in the C-terminal domain of GAT1 that are responsible for supporting GAT1 export from the endoplasmic reticulum and for putting GAT1 under the control of the exocyst (Farhan et al., 2004; Moss et al., 2009). In addition to these, the MAGUK protein Pals1 is co-expressed with GAT1 in COS7 cells and contributes to stabilize GAT1 on the cell membrane (McHugh et al., 2004).

### Regulation of GABA Uptake

The control of GABA uptake can be expressed through changes in the rate with which GABA transporters are trafficked and redistributed in the plasma membrane, with consequences on the number of transporter molecules available for binding extracellular GABA, or by modulating the biophysical properties of GABA transporters (e.g., *V*_*max*_, *k*_*m*_, etc.). Neurons regulate the surface expression of GAT1 in parallel with that of extracellular neurotransmitter levels. GABA transporters interact with the SNARE proteins syntaxin 1A and Munc-18 through molecular interactions modulated by PKC (Beckman et al., 1998; Deken et al., 2000; Geerlings et al., 2001; Horton and Quick, 2001). These transporters are associated with presynaptic vesicles similar to synaptic vesicles, capable of undergoing clathrin-mediated internalization and endosomal sorting (Barbaresi et al., 2001). Despite being morphologically similar to synaptic vesicles, and despite the fact that they can be released at similar rates in a calcium dependent manner, they lack synaptophysin and vesicular GABA transporters (Deken et al., 2003). It has been suggested that these GABA transporter-containing vesicles might represent a population of endocytic or exocytic vesicles acting as cargos for the assembly of synaptic domains distinct from the active zone (Deken et al., 2003).

PKC modulation is important to change the number of functional GABA transporters expressed on the plasma membrane (Corey et al., 1994; Quick et al., 1997). In *Xenopus laevis* oocytes, PKC activation with PMA promotes GAT1 translocation to the cell membrane at low basal GAT1 expression levels, but this effect is not detected at high levels of expression of GAT1. The rate of GABA uptake can also be altered by PKC (Corey et al., 1994; Quick et al., 1997). Accordingly, inhibiting PKC reduces GABA uptake through a reduction of *V*_*max*_ (not *k*_*m*_), whereas inhibiting protein phosphatase 2B increases it. These forms of PKC-dependent modulation differ in cultured neurons and isolated nerve terminals, where surface expression of GAT1 is decreased by PKC dependent phosphorylation (Beckman et al., 1999; Wang and Quick, 2005; Cristovao-Ferreira et al., 2009).

Another protein kinase, PKA, stimulates GABA transport via GAT1 and activates a GAT1-mediated cationic current during opioid withdrawal (Bagley et al., 2005). This enhanced PKA signaling contributes to increase neuronal action potential firing rates in opioid-sensitive PAG neurons during opioid withdrawal (Bagley et al., 2005).

In the area CA1 of the rat hippocampus, chronic stimulation of cannabinoid receptors CB1/2 reduces GAT1 gene expression (Higuera-Matas et al., 2012). Conversely, an endogenous agonist of endocannabinoid receptors, 2-arachidonoylglycerol (2-AG), increases GABA uptake (Romero et al., 1998; Venderova et al., 2005). CB1 receptors have been suggested to interact or co-localize with β_2_ adrenergic receptors (Hudson et al., 2010), the activation of which also increases GAT1 expression and GABA uptake (Martins et al., 2018). The ability of β_2_ adrenergic receptors to promote GABA uptake (and that of β_1_ adrenergic receptors to inhibit it) are mediated by a PKA pathway controlled by cannabinoid receptors, because the modulation of GABA update by adrenergic receptors is inhibited by the cannabinoid receptor agonist WIN55,212-2 (Martins et al., 2018).

In astrocytes, GABA uptake via GAT1, not GAT3, is modulated by neurotrophic factors like BDNF (Vaz et al., 2011). This is due to the ability of BDNF to inhibit dynamin/clathrin-dependent constitutive internalization of GAT1, effectively increasing the lifetime of GAT1 on the cell membrane. The effect of BDNF is mediated by activation of a truncated form of the TrkB receptor, is coupled to a PLC-γ/PKC-δ and ERK/MAPK pathway, and requires activation of adenosine A2A receptors (Vaz et al., 2011). The cross talk between A2A receptors and BDNF is likely due to the fact that activation of A2A receptors activates TrkB and induces its translocation to lipid rafts (Tebano et al., 2008; Assaife-Lopes et al., 2010). This type of modulation differs in neurons, where BDNF can still inhibit GABA uptake via GAT1 in conjunction with A2A receptors, but here the BDNF modulation persists in the presence of A2A receptor antagonists or upon removal of extracellular adenosine (Vaz et al., 2008).

### Physiological Roles of GABA Transporters

Genetic variants in the solute carrier family 6 member 1 (SLC6A1) gene, encoding GAT1, are associated with various neurodevelopmental disorders, including epilepsy with myoclonic atonic seizures, autism spectrum disorder and intellectual disability (Bhat et al., 2020; Goodspeed et al., 2020). Knockout mouse models of GAT1, as well as GAT1 inhibitors, have shown a range of physiological effects that indicate that GAT1, through its exquisite regulation of GABA in the brain, may be an interesting target for therapies for neuropsychiatric diseases (Salat and Kulig, 2011; Egawa and Fukuda, 2013; Bhat et al., 2020). GAT1 inhibitors, such as tiagabine, NO-711 and DDPM-2571, have anti-seizure, antinociceptive, antiallodynic and anxiolytic properties (Laughlin et al., 2002; Todorov et al., 2005; Pakulska, 2007; Xu et al., 2008; Salat et al., 2017). Loss of GAT1 in the *nucleus accumbens* has also been observed in mice treated with chronic social defeat stress paralleling observations in patients with major depressive disorder (MDD) (Heshmati et al., 2020).

During epileptic seizures, the ionic gradient that typically supports GABA uptake from the extracellular space can be dissipated or even inverted, promoting GABA release through the reversed activity of GABA transporters like GAT3 (Raiteri et al., 2002; Wu et al., 2003; Richerson and Wu, 2004; Kinney, 2005). Under these conditions, the reversal of GABA uptake could provide a useful mechanism to curtail seizure propagation. With some exceptions (Xie et al., 2017), single nucleotide polymorphisms in SLC6A11, the gene encoding human GAT3, have been detected in patients with antiepileptic drug resistance (Kim et al., 2011). It is possible that a dysfunction of GAT3 could alter both phasic and tonic GABAergic transmission in the epileptic brain.

GATs regulate neurotransmission in other complex ways. As mentioned above, substrate transport through GAT elicits an inward current that can directly excite neurons (Bagley et al., 2005, 2011). GAT1 also generates a sodium-dependent capacitive current that can also contribute to shunting (Mager et al., 1993). Efflux of GABA into the extracellular space contributes to tonic currents mediated by GABA_*A*_ receptors in hippocampal neurons (Wu et al., 2007) and glia (Barakat and Bordey, 2002). Tonic currents are critical in neurodevelopment (Egawa and Fukuda, 2013) and are increased in pathological conditions, such as inflammation (Tonsfeldt et al., 2016). Regulation of GAT activity and trafficking has a dramatic effect on tonic currents, especially those mediated by extrasynaptic GABA_*A*_ receptors (Scimemi et al., 2005; Scimemi, 2014a,b). In addition to controlling integration of inputs onto GABAergic neurons, these tonic currents control the coincidence detection window of excitatory inputs onto pyramidal neurons (Sylantyev et al., 2020) and contribute to regulation of dopamine release in the dorsal striatum (Roberts et al., 2020). GAT3 activity in astrocytes regulates release of ATP and adenosine that contributes to heterosynaptic depression in the hippocampus (Boddum et al., 2016), highlighting an additional mechanism for regulating synaptic activity.

## Dopamine Uptake: One Transporter, Three Currents, Multiple Regulation Sites

**FIGURE 3:**
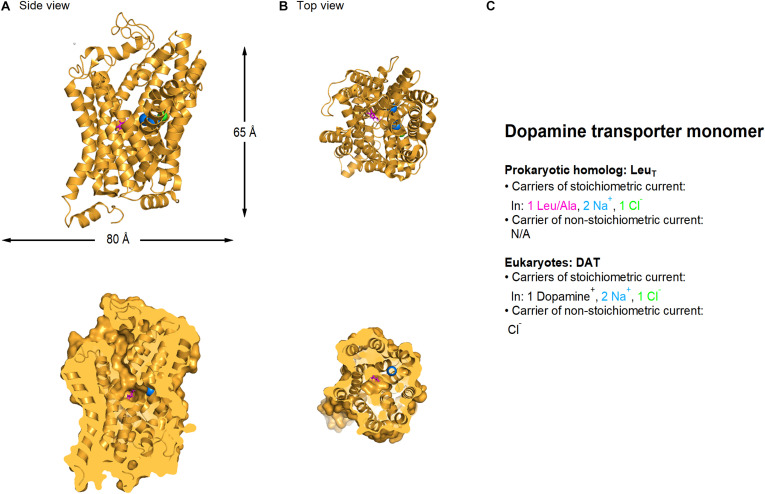
Crystal structure of *Drosophila melanogaster* dDAT dopamine transporter (PDB: 4XP1). **(A)** Side view of the *Drosophila* dopamine transporter parallel to the membrane, using a ribbon (*top*) and a surface representation sliced through the center of the transporter (*bottom*). **(B)** As in A, viewed from the extracellular side of the membrane. L-Dopa (*magenta*), sodium (*blue*) and chloride ions (*green*) are represented as spheres. **(C)** Summary of the main feature of the stoichiometric and non-stoichiometric current mediated by LeuT (prokaryotic dopamine transporter homolog) and DAT transporters in eukaryotes, respectively. Image generated using The PyMOL Molecular Graphics System (Version 2.3.2; Schrödinger, 2010).

Dopamine uptake via the membrane transporter DAT is stoichiometrically coupled to the co-transport of two Na^+^ and one Cl^–^ ion (Figure 3) (Krueger, 1990; McElvain and Schenk, 1992; Gu et al., 1994; Penmatsa et al., 2013, 2015). Voltage-clamp recordings of DAT-mediated currents in *Xenopus* oocytes, however, show that the mean net charge to dopamine ratio is significantly larger than the one predicted by the stoichiometric coupling ratio (Sonders et al., 1997; Ingram et al., 2002). This discrepancy can be accounted for by the fact that DAT, like glutamate and GABA transporters (as well as serotonin and norepinephrine transporters; Bruns et al., 1993; Cammack et al., 1994; Mager et al., 1994; Galli et al., 1995; Jayanthi et al., 2002), also mediates two stoichiometrically uncoupled conductances: one that requires dopamine binding to the transporter, and one that does not and is constitutively active. For simplicity, we refer to them as the uncoupled and the leak conductance, respectively.

It is interesting to note that the concentration of dopamine required for half-maximal transport is ∼580 nM, whereas the one required for activation of the uncoupled current is only ∼35 nM (Ingram et al., 2002). The extracellular concentration of dopamine in the brain has been known to vary on a sub-second time scale due to phasic firing of dopaminergic neurons, and to be modulated by administration of substances of abuse. Until recently, fast-scan cyclic voltammetry (FSCV) has been considered the technique that provides the best combination of temporal resolution, sensitivity and chemical selectivity (Roberts et al., 2013), but even in this case it has been traditionally used to obtain relative, as opposed to absolute values of dopamine concentrations in the brain. In 2018, the development of cellular-scale probes have enabled stable recording of sub-second extracellular dopamine oscillations (Schwerdt et al., 2018). Although the recorded levels of dopamine were specific for each mouse, they ranged between 40–450 nM (Schwerdt et al., 2018). This means that the uncoupled DAT current is likely to be a main contributor to cell excitability and neurotransmitter release in DAT-expressing neurons. The uncoupled DAT-mediated current is carried by chloride and, surprisingly, its activation increases cell excitability in cultured dopaminergic neurons (Ingram et al., 2002; Carvelli et al., 2004).

In physiological conditions, the leak current is carried by K^+^ and perhaps H^+^ ions. This leak current is voltage-dependent, outward-rectifying and reverses at ∼–10 mV (Sonders et al., 1997). Although its activation does not require ion or agonist binding to the transporter, it can be blocked by dopamine and other DAT ligands. The ability of substrates to block the leak current is not consistently observed among other members of the Na^+^/Cl^–^-dependent cotransporter family, with the exception of GAT1 (Mager et al., 1994; Cammack and Schwartz, 1996; MacAulay et al., 2002; Kanner, 2003) and the serotonin (Mager et al., 1994) and norepinephrine transporters (Galli et al., 1995). These findings identify multiple mechanisms through which dopamine transporters can change the membrane potential and the signaling properties of neurons through mechanisms that are distinct from its ability to take up dopamine from the extracellular space.

A summary of current studies on modulation of dopamine transporters listed in PubMed is provided in Table 3.

**TABLE 3:** Regulating factors of dopamine transporter Uptake and expression.

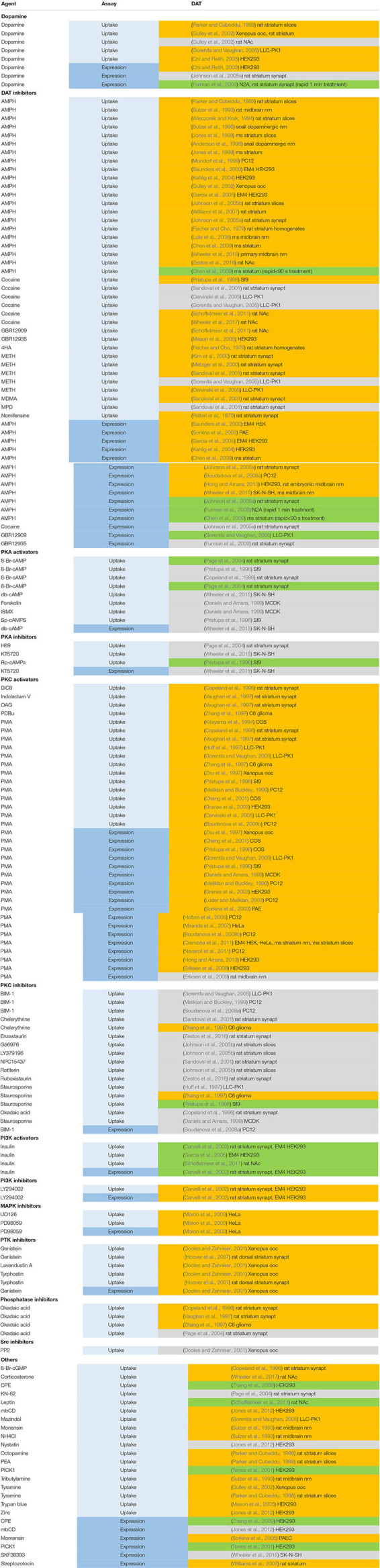

*The table provides a summary of the results obtained in the literature regarding the mechanisms controlling the rate of dopamine uptake and its expression in a variety of animal models and expression systems. The color coding of each cell and abbreviations are as described for Table 1. *NAc*, *nucleus accumbens; AMPH*, amphetamine; *CPE*, carboxypeptidase E; *4HA*, 4-Hydroxyamphetamine; *IBMX*, 3-isobutyl-1-methylxanthine; *METH*, methamphetamine; *MPD*, methylphenidate; *PEA*, phenylethylamine; *PP2*, protein phosphatase 2.*

### The Pharmacology of Dopamine Transporters

DAT is a target of psychostimulants like cocaine and amphetamine. Cocaine binds to DAT and increases extracellular dopamine levels by blocking its transport activity (Chen and Reith, 2000; Norregaard and Gether, 2001; Goldberg et al., 2003; Torres et al., 2003). In contrast, amphetamine is a substrate for this transporter (Kahlig et al., 2005). The inward transport of amphetamine increases the number of inward-facing transporter binding sites, which results in an increased rate of dopamine efflux through what at first sight may look like an exchange process (Sulzer et al., 1995; Kahlig et al., 2005). In reality, this phenomenon may be more complex, as evidenced by the fact that N-terminus phosphorylation of DAT alters only the amphetamine-induced dopamine efflux without altering dopamine uptake (Khoshbouei et al., 2004). Dopamine and amphetamine are both substrates for DAT, and compete with each other for binding to the transporter (Sulzer et al., 1993; Hoffman et al., 1998). They can both trigger dopamine efflux through reversed uptake, by inverting the electrochemical gradient for Na^+^ and Cl^–^ (Sitte et al., 1998). Whereas dopamine inhibits the DAT channel-like behavior, amphetamine activates it. This effect is not due to differences in the ability of dopamine and amphetamine to change the intracellular Na^+^ concentration, because it can still be detected in outside-out patches, where the intracellular Na^+^ concentration is controlled (Kahlig et al., 2005).

### The Surface Mobility and Cellular Distribution of Dopamine Transporters

Dopamine transporters are monoamine neurotransmitter transporters located in the pre-synaptic terminal of dopaminergic neurons, away from the synaptic area (Nirenberg et al., 1997). These cells are located in the ventral tegmental area and *substantia nigra*, project to the cingulate and medial pre-frontal cortex, *nucleus accumbens*, olfactory tubercle, lateral habenula, and *striatum*. Through their axonal projections, dopaminergic neurons control motivation, reward reinforcement and movement, in addition to attention, planning and memory. Therefore, the regulation of DAT function has extensive functional implications for all these behaviors. It is now evident that the role of DAT is not limited to regulating the extracellular concentration of dopamine but is also involved in the homeostatic maintenance of presynaptic function (Torres et al., 2003). Like other membrane proteins, DAT is associated with intracellular proteins that ensure the appropriate location of the transporter in specific domains of the cell membrane at a given time. Accordingly, DAT has been shown to bind to the SNARE protein complex syntaxin 1A (Lee et al., 2004), the PDZ domain protein PICK1 (Torres et al., 2001; Bjerggaard et al., 2004), CaMKII (Fog et al., 2006), and the multiple-LIM-domain-containing adaptor protein HIC-5 (Carneiro et al., 2002).

DAT is equally distributed in and out of rafts (Foster et al., 2008), nanometer-wide and temporally dynamic lipid microdomains enriched in cholesterol, sphingolipids and glycosylphosphatidylinositol (GPI)-anchored proteins (Hancock, 2006). Structural studies of the *Drosophila melanogaster* DAT (dDAT) reveal that cholesterol can bind in a crevice formed by transmembrane domains 5, 7, and 1a, which is thought to prevent the conformational changes required for the transporter to transition from outward-facing to inward-facing (Penmatsa et al., 2013, 2015). This is in agreement with functional studies that show DAT can be regulated by cholesterol, due to its ability to promote an outward-facing conformation of the transporter (Hong and Amara, 2010; Jones et al., 2012).

By using Fluorescence Correlation Spectroscopy (FCS) and FRAP, Adkins et al. (2007) measured the surface diffusion coefficient of DAT in two types of cells: HEK293 and N2a cells. Due to differences in laser beam waist and sampling areas between FRAP and FCS, FCS is better suited to detect fast protein movements within a confined domain, whereas FRAP allows to detect long-range diffusion between domains in the membrane (Adkins et al., 2007). In HEK293 cells, DAT diffuses with a diffusion coefficient of 3.6 × 10^–9^ cm^2^/s, consistent with a relatively freely diffusible protein. This diffusion coefficient is lower in N2a cells, where DAT is partially immobilized (*D* < 10^–10^ cm^2^/s). This difference is likely due to the existence of cell-specific direct or indirect interactions between DAT and cytoskeletal proteins or with membrane rafts. Accordingly, single particle tracking studies confirm that the median diffusion coefficient for DAT is 1.6 × 10^–10^ cm^2^/s in Flp-In 293 cells (Kovtun et al., 2015).

Overall, these findings indicate that DAT transporters diffuse substantially slower and in a more confined manner than the glutamate transporter GLT-1, probably because of the presence of different types of protein interactions in different membrane transporters or because of the presence/lack of specific interacting substrates (Murphy-Royal et al., 2015).

### Transcriptional, Translational, and Post-translational Regulation of Dopamine Transporters

The human DAT gene was first cloned in 1991, and it is localized to chromosome 5p15.3 and has a single transcriptional start site (Kilty et al., 1991; Vandenbergh et al., 1992a,b). DAT has a half-life of about 2 days on the cell membrane, suggesting the existence of dynamic processes of transcriptional and translational regulation (Kimmel et al., 2000; Kahlig and Galli, 2003). Transcription factors like *Nurr1* and *Pitx3* have an expression pattern that match that of dopaminergic neurons, and are known to be crucial for the development, survival and maintenance of midbrain dopaminergic neurons (Lee et al., 2010; Rodriguez-Traver et al., 2016; Salemi et al., 2016). Accordingly, disrupting the *Nurr1* gene alters the development of dopaminergic neurons (Zetterstrom et al., 1997; Castillo et al., 1998; Saucedo-Cardenas et al., 1998). *Nurr1* binds with high-affinity to an NGFI-B responsive element within the promoter regions of DAT and other dopamine-related genes (Sacchetti et al., 1999). The ability of *Nurr1* to increase DAT expression, however, relies on mechanisms that are independent of the NGFI-B responsive element. There are a variety of sequence motifs identified through *in silico* studies, which point to the fact that DAT can be regulated epigenetically via DNA methylation and histone acetylation *in vitro* and *in vivo* (Wang et al., 2007). Like other housekeeping genes, the DAT gene lacks conserved TATA and CAAT boxes (confirming that DAT is susceptible to regulation by histone acetylation), and its core promoter is GC-rich (confirming that DAT expression can be regulated via DNA methylation) (Choi and Kim, 2008).

These discoveries prompted a number of biochemical and mutagenesis studies on heterologous expression systems, which led to the identification of key structural features and functional domains of the transporter. A major breakthrough occurred when the first X-ray crystal structure of the bacterial leucine transporter LeuT_*Aa*_, which shares 20–25% homology with all monoamine transporters was solved at 1.65 Å resolution (Yamashita et al., 2005). This was followed by the structure of *Drosophila melanogaster* DAT, which shares 50–55% homology with monoamine transporters (Penmatsa et al., 2013, 2015; Wang et al., 2015). Together, these studies suggest that all monoamine transporters have 12 α-helix spanning domains and an alternating access substrate translocation mechanism (Kristensen et al., 2011). Despite the structural similarity of the core transmembrane regions of all monoamine transporters, their extracellular loops, N- and C-termini differ significantly in length and sequence (Kristensen et al., 2011). This is important because these regions are the site of post-translational modifications and can be the site of protein–protein interactions that control transporter localization, stability and activity (Aggarwal and Mortensen, 2017). Post-translational modifications, binding partner interactions, modulation by cholesterol and membrane raft associations are all capable of modulating DAT activity and ultimately dopamine clearance from the extracellular space.

The N-terminus of the DAT protein is subject to phosphorylation and ubiquitination (Karam and Javitch, 2018). Although five serine residues at positions 2, 4, 7, 12, 13 have been identified as targets for phosphorylation by PKC, the only verified phosphorylation site is Ser7 (Moritz et al., 2013). The localization of these sites at the distal end of a long and flexible domain suggests that these residues may be also regulated by partner interactions, but these effects have not been demonstrated. A second verified phosphorylation site is Thr53, which is followed by a Pro residue, making it specific for proline-directed kinases such as ERK. Thr53 is also flanked by an SH3 domain, which is a ligand for protein scaffolding (Saksela and Permi, 2012). In between these two phosphorylation sites, Lys27 is a residue that undergoes ubiquitination catalyzed by the ubiquitin E3 ligases *Nedd4-2* and *Parkin*. This modification is increased by PKC activation and contributes to promote DAT endocytosis (Hong and Amara, 2010; Vina-Vilaseca and Sorkin, 2010). The N-terminal domain also contains binding sites for the regulatory partners syntaxin 1 (res 1–33, which reduces dopamine uptake) and D2 dopamine receptors (res. 1–15) (Lee et al., 2007; Binda et al., 2008; Carvelli et al., 2008). On the C-terminal domain of the DAT protein, Cys580 provides an *S*-palmitoylation site, and a FREK motif at residues 587–590 binds the small Ras-like GTPase Rin1 and contributes to PKC-mediated endocytosis (Boudanova et al., 2008b; Navaroli et al., 2011). Other regulatory domains in the C-terminal region include binding sites for CaMK (res. 612–617) and α-synuclein (res. 606–620) (Lee et al., 2001; Moszczynska et al., 2007). The C-terminal domain also contains a PDZ domain-binding sequence, where interactions with scaffolding proteins like PICK1 occur. Notably, these interactions are neither necessary nor sufficient for surface targeting of DAT, and have yet unidentified functional consequences (Bjerggaard et al., 2004). Other members of the “DAT interactome” include PP2Ac (Baumann et al., 2000), Hic-5 (Carneiro et al., 2002), RACK (Lee et al., 2004), D_2_ dopamine receptors (Bolan et al., 2007), flotillin-1 (Cremona et al., 2011), Rin (Navaroli et al., 2011), and the k-opioid receptor (Kivell et al., 2014; Bermingham and Blakely, 2016).

The regulatory mechanisms described so far make it easy to spot that protein kinases like PKC, one of the best characterized regulatory proteins for DAT, can regulate dopamine uptake through a variety of processes, including an endocytotic mechanism driven by the phosphorylation of DAT accessory proteins, a kinetic down-regulation mediated by Ser7 phosphorylation, and an increased dopamine efflux mediated by altered surface transporter activity. Together, these modifications allow PKC to reduce dopamine uptake. The actions of PKC are opposite to those induced by palmitoylation, which lead to reduced DAT degradation (Chen et al., 2013). It is important to keep in mind that the primary sites of PKC-stimulated phosphorylation are the membrane rafts, the microdomains rich in cholesterol and sphingolipids. Therefore, changing PKC-mediated phosphorylation of DAT can affect its raft distribution and protein interactions. In contrast to PKC, ERK provides a tonic mechanism to increase dopamine uptake perhaps via phosphorylation of Thr53 (Foster et al., 2012). In addition to PKC and ERK, there is a host of other kinases that are capable of regulating DAT activity, including PKA, PKG, CaMKII, MAPK, PI3K/Akt and tyrosine kinases. PKCβ may also be involved in the mechanism of D_2_ receptor regulation of DAT by ERK (Chen et al., 2013).

### Physiological Roles of Dopamine Transporters

DAT regulates extracellular concentrations of the neuromodulator dopamine and thus, subsequent activation of dopamine receptors that can enhance or inhibit neurons. DAT is the target of psychoactive and psychotherapeutic drugs such as methylphenidate and amphetamines that are used in treatment of attention deficit and hyperactivity disorder (ADHD) symptoms, as well as drugs of abuse (Schmitt et al., 2013; German et al., 2015). Genetic variants of SLC6A3, the gene encoding DAT in humans, have been identified in patients with neuropsychiatric, neurodevelopmental and neurodegenerative disorders (Mazei-Robison et al., 2005; Serretti and Mandelli, 2008; Mick and Faraone, 2009; Sakrikar et al., 2012; Bowton et al., 2014; Hansen et al., 2014; Mergy et al., 2014; Herborg et al., 2018; Campbell et al., 2019; DiCarlo et al., 2019). Studies of these variants have observed deficits in uptake, transporter-associated currents, trafficking and regulation (Gowrishankar et al., 2014). Further studies of these mutants should give more insight into the complex regulation of dopamine-modulated circuits. An intriguing new area is the role that DAT plays in transport of ligands that act on the trace amine-associated receptor subtype 1 (Taar1), an intracellularly localized G protein-coupled receptor that can, in turn, regulate internalization of both the DAT and EAAT3 in dopaminergic neurons (Underhill et al., 2014, 2019), enhancing extrasynaptic glutamate signaling (Li et al., 2017). Taar1 is known to regulate monoaminergic signaling and dysregulation of TAAR1 signaling may play important roles in neuropsychiatric disorders (Schwartz et al., 2018; Dodd et al., 2020).

## Conclusion

Transporters for excitatory, inhibitory, and modulatory neurotransmitters like glutamate, GABA and dopamine are complex molecular machines that do much more than act as vacuums that clear neurotransmitters out of the extracellular space. This complexity arises in part from the core biophysical properties of these transporters, which are capable of generating different types of ionic currents. The additional levels of complexity comes from the fact that their residence time on the membrane, their trafficking from intracellular compartments and their kinetics can be modulated at different levels, including transcriptional, epigenetic, translational and post-translational levels. All these forms of modulation can change across species, cell types and brain regions. Since these regulatory mechanisms also change over time, their efficacy likely follows the metabolic state of a given neuron or astrocyte. The knowledge accumulated over the last few years and the growth of novel experimental approaches will undoubtedly provide new insights into cell-specific changes in the way the activity of neurotransmitter transporters can control cell excitability and metabolism across the brain.

## Author Contributions

RMR and SLI wrote the manuscript. AS wrote the manuscript and coordinated the investigative team. All authors contributed to the article and approved the submitted version.

## Conflict of Interest

The authors declare that the research was conducted in the absence of any commercial or financial relationships that could be construed as a potential conflict of interest.
